# Curcumol repressed cell proliferation and angiogenesis *via* SP1/mir-125b-5p/VEGFA axis in non-small cell lung cancer

**DOI:** 10.3389/fphar.2022.1044115

**Published:** 2022-11-18

**Authors:** Changju Ma, Xiaojuan Tang, Qing Tang, Shiyan Wang, Junhong Zhang, Yue Lu, Jingjing Wu, Ling Han

**Affiliations:** ^1^ The Postdoctoral Research Station, Guangdong Provincial Hospital of Chinese Medicine, The Second Clinical Medical College, Guangzhou University of Chinese Medicine, Guangzhou, China; ^2^ GuangDong Academy of Traditional Chinese Medicine, Research Team of Bio-molecular and System Biology of Chinese Medicine, Guangdong Provincial Hospital of Chinese Medicine, Guangzhou, China; ^3^ Central Laboratory, Hunan Provincial Hospital of Integrated Traditional Chinese and Western Medicine, The Affiliated Hospital of Hunan Academy of Traditional Chinese Medicine, Changsha, China; ^4^ Guangdong Provincial Key Laboratory of Clinical Research on Traditional Chinese Medicine Syndrome, Guangzhou, China; ^5^ Guangdong-Hong Kong-Macau Joint Lab on Chinese Medicine and Immune Disease Research, Guangzhou University of Chinese Medicine, Guangzhou, China; ^6^ Department of Emergency, The Affiliated TCM Hospital of Guangzhou Medical University, Guangzhou, China; ^7^ State Key Laboratory of Dampness Syndrome of Chinese Medicine, The Second Affiliated Hospital of Guangzhou University of Chinese Medicine, Guangzhou, China

**Keywords:** curcumol, NSCLC, Sp1, miR-125b-5p, VEGFA, angiogenesis, tumor microenvironment

## Abstract

NSCLC (non-small cell lung cancer) is one of the most common and lethal malignant tumors, with low 5-year overall survival rate. Curcumol showed antitumor activity in several cancers, but evidence about its effect on NSCLC remains unclear. In the present study, we found that Curcumol markedly inhibited NSCLC cells proliferation, migration and invasion. Endothelial cells are an important part of tumor microenvironment. Tube formation assay and wound healing assay indicated that A549 derived conditioned medium affected HUVECs (human umbilical vein endothelial cells). Mechanistically, Curcumol downregulated the expression of SP1 (specificity protein 1) while upregulated miR-125b-5p, followed by decreasing VEGFA expression in NSCLC cells. Furthermore, overexpression of SP1 partially reversed the inhibitory effect of Curcumol on A549 and H1975 cell viability and VEGFA expression. Inhibition of miR-125b-5p presented similar effect. Interestingly, there was mutual modulation between SP1 and miR-125b-5p. Collectively, our study revealed that Curcumol inhibited cell growth and angiogenesis of NSCLC *in vitro* and *in vivo*, possibly through SP1/miR-125b-5p/VEGFA regulatory mechanism. These findings may provide effective therapy strategies for NSCLC treatment.

## Introduction

Lung cancer is one of the most common cancers and its incidence rate of men and women in the world is in the second place. Moreover, lung cancer mortality ranks first among all kinds of cancers. NSCLC (non-small cell lung cancer) accounts for about 85% of lung cancer, of which lung adenocarcinoma is the most common subtype (Torre et al., 2016; Testa et al., 2018; Barta et al., 2019). About 70% of NSCLC are locally advanced or metastatic at the time of diagnosis. In the past decade, the 5-year overall survival rate of patients with advanced NSCLC was less than 5% (Arbour and Riely, 2019). Therefore, emerging therapies have been tried to select more effective ways to inhibit lung cancer occurrence and development, prolong the survival period of patients, and improve their quality of life.

Curcumol (used in Chinese medicine), a bioactive sesquiterpenoid, extracted from Rhizoma Curcumae, has been reported to present antitumor effects in various cancers (Yan et al., 2018; Zhang et al., 2018; Huang et al., 2020; Wang et al., 2020). Curcumol treatment produced reactive oxygen species (ROS) and inactivated Akt/GSK3β/cyclin D1 pathway, which led to cell cycle arrest in G0/G1 phase, thus preventing colon cancer cells growth (Wang et al., 2018). Curcumol attenuated melanoma development by promoting miR-152-3p expression, suppressing ERK/NF-κB signaling and c-MET/PI3K/AKT signaling pathway (Ning et al., 2020). Curcumol could also inhibit NSCLC cell progression. For example, Curcumol triggered cell death in a caspases-independent mitochondrial apoptosis pathway in ASTC-a-1 cells, a subtype of human lung adenocarcinoma cells (Zhang et al., 2011). However, the study of curcumol effect on the antitumor activity is limited, especially when it comes to lung cancer.

Tumor microenvironment, where tumor cells located, consists of diverse components, such as tumor cells, capillaries, stromal cells, macrophages (Plate et al., 2012). Angiogenesis, one of the well-known ten characteristics of tumors, refers to the formation of novel capillary blood vessels from already existing ones and some capillary venules in the tumor microenvironment. It requires vascular endothelial cell proliferation, matrix degradation, migration and branching to evolve into new tubes (Li et al., 2019). Tumor angiogenesis played rather crucial roles in tumor cells proliferation, migration, invasion and metastasis as it provided nutrition and oxygen for tumor cells growth. Angiogenesis targeting was proved to be valid for tumor treatment. Anti-angiogenic agents have been commonly used in clinic in recent years (Hanahan and Weinberg, 2011; Abels et al., 2019). For example, Axitinib, a multi-target tyrosine kinase inhibitor, as an oral, potent antiangiogenic drug, mainly targets VEGFR-2 (vascular endothelial growth factor receptor-2) tyrosine kinase domain (Canu et al., 2011). VEGFA (vascular endothelial growth factor A), is known as a potent mediator of pathological and physiological angiogenesis and a validated target for anti-angiogenesis treatment clinically (Apte et al., 2019). However, many anti-angiogenesis agents also caused endothelial cells dysfunction and exhibited drug resistance (Simons et al., 2016). Therefore, less toxic and more effective agents in anti-tumor angiogenesis are required.

SP1 (specificity protein 1) is a well-known transcription factor, which involves in a variety of biological processes and has been proven relevant with cell growth, apoptosis, differentiation and carcinogenesis (Zhang et al., 2020). SP1 activates many cellular genes transcription that possess putative CG-rich binding sites in the promoters, leading to enhanced or repressed gene expression (Vizcaíno et al., 2015). High levels of SP1 protein were defined to be a negative prognostic factor in many types of cancer (Jhu et al., 2021; Zhang et al., 2021). For example, in lung cancer, higher SP1 expression with lower PDSS2 expression was found significantly correlated with poor prognosis (Hu et al., 2019).

MicroRNAs (also referred as miRNAs), a class of small non-coding RNAs with around twenty nucleotides in length, can regulate gene expression level by binding to the 3′UTR (3′ untranslated region) of mRNAs, resulting in mRNAs degradation or translation blockade. Notably, miRNAs play an important role in approximately all biological processes, including tumorigenesis (Wang et al., 2021). Among plenty of miRNAs, dysregulation of miR-125b-5p were demonstrated in several cancers, such as bladder cancer (Liu et al., 2020), breast cancer (Li et al., 2018), colon cancer (Park et al., 2020), hepatocellular carcinoma (Hua et al., 2019). Nevertheless, the role of miR-125b-5p in NSCLC has yet been explored sufficiently.

In the present study, we designed both *in vitro* and *in vivo* assays to explore potential underlying mechanisms by which Curcumol conveyed the inhibitory effects on NSCLC cell proliferation and angiogenesis. Our results showed that Curcumol decreased VEGFA expression through reciprocal interaction of SP1 and miR-125b-5p, leading to cell growth inhibition and impaired angiogenesis. Collectively, our findings suggest that Curcumol act as a potent angiogenesis modulator and may provide novel insights into therapeutic strategy for lung cancer.

## Materials and methods

### Cell lines and reagents

A549, H1975, BEAS-2B, 293T cells and HUVECs were purchased from Chinese Academy of Sciences Cell Bank of Type Culture Collection (Shanghai, China), authenticated for morphology, genotypes, drug response and absence of *mycoplasma*. A549, H1975, BEAS-2B were cultured with RPMI-1640 medium (Gibco, NY, United States). 293T cells were cultured with DMEM medium (Gibco, NY, United States). Both kinds of medium were supplemented with 10% fetal bovine serum (Gibco), 100 U/ml penicillin and 100 μg/ml streptomycin (Gibco). HUVECs were cultured with Endothelial Cell Growth Medium (Lonza, Basel, Switzerland). A549-Luc cells containing luciferase reporter gene were constructed by Land Biological Technology (Guangzhou, China), screened with 200 μg/ml Geneticin (G-418) Sulfate (Life Technologies, United States). All the cells were placed in 37°C, 5% CO_2_ incubator. Curcumol was obtained from Chengdu Must Bio-technology (purity 99%, Chengdu, China). Axibinib was purchased from MedChemExpress (purity 99.94%, NJ, United States).

### MTT assay

The 3- (4,5-dimethylthiazol-2-yl) -2,5-diphenyltetrazolium bromide (MTT) assay was conducted to detect the cell viability. A549, H1975, BEAS-2B cells (4000 cells/well) were seeded into 96-well plates, then treated with indicated doses of Curcumol for 24 h, 48 h, and 72 h. Next, cells were cultured with MTT solution (5 mg/ml) at 37°C for at least 4 h, followed by adding DMSO (dimethyl sulfoxide). Microplate reader (Synergy H1, United States) was used to measure OD (optical density) at 490 nm. The experiments were performed independently five times (n = 5) in triplicate.

### EdU assay

EdU (5-Ethynyl-2′-deoxyuridine) assay kit (RIBOBIO, Guangzhou, China) was applied to evaluate cell proliferation. A549 and H1975 cells were treated with Axitinib (4 μM) or Curcumol (400 μM) for 24 h, then incubated with EdU for 2 h, fixed with 4% paraformaldehyde for 30 min, incubated in 0.2% TritonX-100 for 10 min, added with 1× Apollo buffer, stained with 1×Hoechest (5 mg/ml). Fluorescence microscope (Eclipse Ti2-E, Japan) was used to capture images.

### Wound healing assay

Cells were seeded in 6-well plates. When cells confluence reached 90%, scratches were made by 200 μL micropipette tip. The wells were gently washed with PBS twice. Then, cells were treated with Axitinb or Curcumol. 0 h and 24 h images were recorded, and relative wound healing rate was calculated by comparing difference of wound width before and after scratching.

### Transwell invasion assay

The invasion assay was carried out by using 24-well Matrigel chambers (BD Biosciences, United States). 2 × 10^5^ A549 cells in 200 μL RPMI-1640 without FBS were seeded in the upper chamber, which was added with Matrigel in advance. 500 μL RPMI-1640 supplemented with 20% FBS was added to the lower chamber. After 24 h administration of Curcumol or Axitinib, cells on the upper surface of the filter were taken away by cotton swab, while cells on the lower surface were immediately fixed with 4% paraformaldehyde and stained with crystal violet.

### Conditioned medium collection

A549 cells were cultured in 6-well plates overnight, then supplemented with Axitinib (4 μM) or Curcumol (200, 400 μM). After 24h, CM (conditioned medium) was harvested by taking supernatant after centrifugation. CM was named as following: 1) Con-CM, 2) Axitinib 4 μM-CM, 3) Curcumol 200 μM-CM and 4) Curcumol 400 μM-CM.

### HUVECs tube formation

HUVECs were seeded into Matrigel-coated 24-well plates at a density of 1 × 10^5^ cells per well and cultured with different conditioned medium as described above for 24 h. Branch points and capillary length were calculated from images by ImageJ software.

### Western blot

Cell total protein was extracted, then resolved on 10% SDS-PAGE, and transferred onto PVDF (polyvinylidene fluoride) membranes. Non-specific binding sites were blocked by 5% non-fat milk for 1 h at room temperature. Subsequently, the membranes cut and probed with corresponding primary antibodies at 4°C overnight, followed by incubation with the secondary antibodies for 2 hours at room temperature. Primary antibodies information is as follows: anti-VEGFR2 (phospho Y951) (ab38473, Abcam), anti-VEGFR2 (ab39638, Abcam), anti-VEGFA (ab46154, Abcam), anti-SP1 (9389S, Cell Signaling Technology, CST for short), anti-β-Tubulin (2128S, CST).

### qRT-PCR assay

Real-time quantitative Polymerase Chain Reaction (qRT-PCR) was performed to detect the expression of VEGFA, SP1 mRNA and miR-125b-5p. Total RNA (1 μg) was reversed to cDNA by using First Strand cDNA synthesis kit (Vazyme, Nanjing, China). A total of 20 μL mixed liquid, containing cDNA, primers and enzyme mixture was added to the PCR 96-well plates for PCR reaction in PCR instrument (ABI ViiA7, United States). The PCR reaction procedure was as follows: 95°C for 30 s, then 95°C for 10 s plus 60°C for 30 s for 40 cycles. GAPDH or U6 was used as endogenous control. Primers sequences were designed as follows:

VEGFA forward: 5′-TAG​AGT​ACA​TCT​TCA​AGC​CGT​C-3′,
VEGFA reverse: 5′-CTT​TCT​TTG​GTC​TGC​ATT​CAC​A-3′;
SP1 forward: 5′-CTG​GTG​GGC​AGT​ATG​TTG​TG-3′,
SP1 reverse: 5′-AAG​CTG​GCA​GAA​CTG​ATG​GT-3′;
GAPDH forward: 5ʹ-CTC​CTC​CTG​TTC​GAC​AGT​CAG​C-3ʹ,
GAPDH reverse: 5ʹ-CCC​AAT​ACG​ACC​AAA​TCC​GTT-3ʹ;
U6 forward: 5′-ATT​GGA​ACG​ATA​CAG​AGA​AGA​TT-3′,
U6 reverse: 5′-GGA​ACG​CTT​CAC​GAA​TTT​G-3′;
miR-125b-5p forward: 5′-ACT​GAT​AAA​TCC​CTG​AGA​CCC​TAA​C-3′,
miR-125b-5p reverse: 5′-TAT​GGT​TGT​TCT​GCT​CTC​TGT​CAC-3′.

### Immunofluorescence staining

A549 cells were seeded on slides (SPL life sciences, South Korea) then transfected with SP1 overexpression vectors (pcDNA3.1-SP1) and pcDNA3.1 negative control vectors (GeneCopoeia, United States), followed by treating with Curcumol for 24 h. MiR-125b-5p mimics, inhibitors and NC were generated by GenePharma (Shanghai, China). Cells were fixed with 4% paraformaldehyde for 10 min and permeabilized for 15 min with 0.3% TritonX-100, then incubated for an hour in blocking buffer (1% Bovine Serum Albumin) at room temperature. Cells were incubated with primary antibodies against VEGFA (ab39250, Abcam) at 4°C overnight. Next day, cells were incubated with Alexa Fluor 488 anti-rabbit antibodies for 1 h. Anti-fluorescence quenching sealing agent containing DAPI (Beyotime, China) were added onto slides, then analyzed by confocal microscopy (ZEISS LSM710, Germany).

### Dual-luciferase reporter assay

The wild-type and mutation-type VEGFA 3′UTR luciferase vectors were synthesized (GeneCopoeia, MD, United States). SP1 promoter vector with Renilla luciferase reporter as internal reference was also generated by GeneCopoeia. Co-transfected these vectors into cells with either miR-125b-5p mimics or inhibitors and corresponding negative control using Liposomal Transfection Reagent (Yeasen, Shanghai, China). Detection of luciferase activities was undergone by using Luc-Pair™ Dual-Luciferase HS Assay Kit (GeneCopoeia MD, United States). Optical density was normalized with Renilla within each sample.

### Immunohistochemistry analysis

Subcutaneous xenograft tumor tissue of nude mice was fixed with 4% formaldehyde for about 24 h before paraffin-embedding, slicing. After antigen retrieval with microwave method, the primary antibody of PCNA (13110S**,** diluted into 1:4000, CST), VEGFA (ab39250, diluted into 1:100, Abcam), SP1 (9389S, diluted into 1:2000, CST), CD31 (3528S, diluted into 1:1000, CST) were all incubated at 4°C overnight, followed by secondary antibody for 1 h. 3,3ʹ- diaminobenzidine (Maixin Biotech, Fuzhou, China) was used as chromogenic agent. Pictures were randomly observed and taken under ×400 magnification from at least five random fields by using upright microscope (BX53+DP72, Olympus Corporation, Japan).

### *In Vivo* study

Animal studies were proceeded according to regulations approved by Guangdong Provincial Hospital of Chinese Medicine Institutional Animal Care and Use Committee (Ethics Approval Number 2020076). BALB/c-nu mice of 6-week-old were provided by Beijing Vital River Laboratory Animal Technology Co., Ltd. 2 × 10^6^ A549-Luc cells (Land Biological Technology, Guangzhou, China) were injected subcutaneously into the right flank, near the axillary fossa region. One week later, mice were randomly assigned into four groups (six mice per group) including Control (saline), Axitinib 20 mg/kg, Curcumol 40 mg/kg, Curcumol 80 mg/kg groups. Saline, Axitinib or Curcumol was given *via* gavage once a day for 25 days. The doses selected referred to other reports (Ma Y. H. et al., 2019; Zuo et al., 2020). Tumor volume was calculated by the formula: volume = (width^2^ × length)/2. Body weight of mice were measured every four days. As for bioluminescence imaging analysis, each goup mice were injected with 10 mg/kg luciferin (Promega, United States). Images were acquired and analyzed by the IVIS-200 Imaging System (Xenogen, Berkeley, United States). After 25 days, all tumor bearing mice were sacrificed, and tumor tissues were isolated and applied for examination of proteins and microRNA expression level.

### Statistical analysis

Statistical analysis was performed by using one-way univariate analysis of variance (ANOVA) with Bonferroni post-hoc test, and values were shown as mean ± standard deviation. Difference with *p* < 0.05 was considered and interpreted to be statistically significant.

## Results

### Curcumol inhibited NSCLC cells proliferation

**FIGURE 1:**
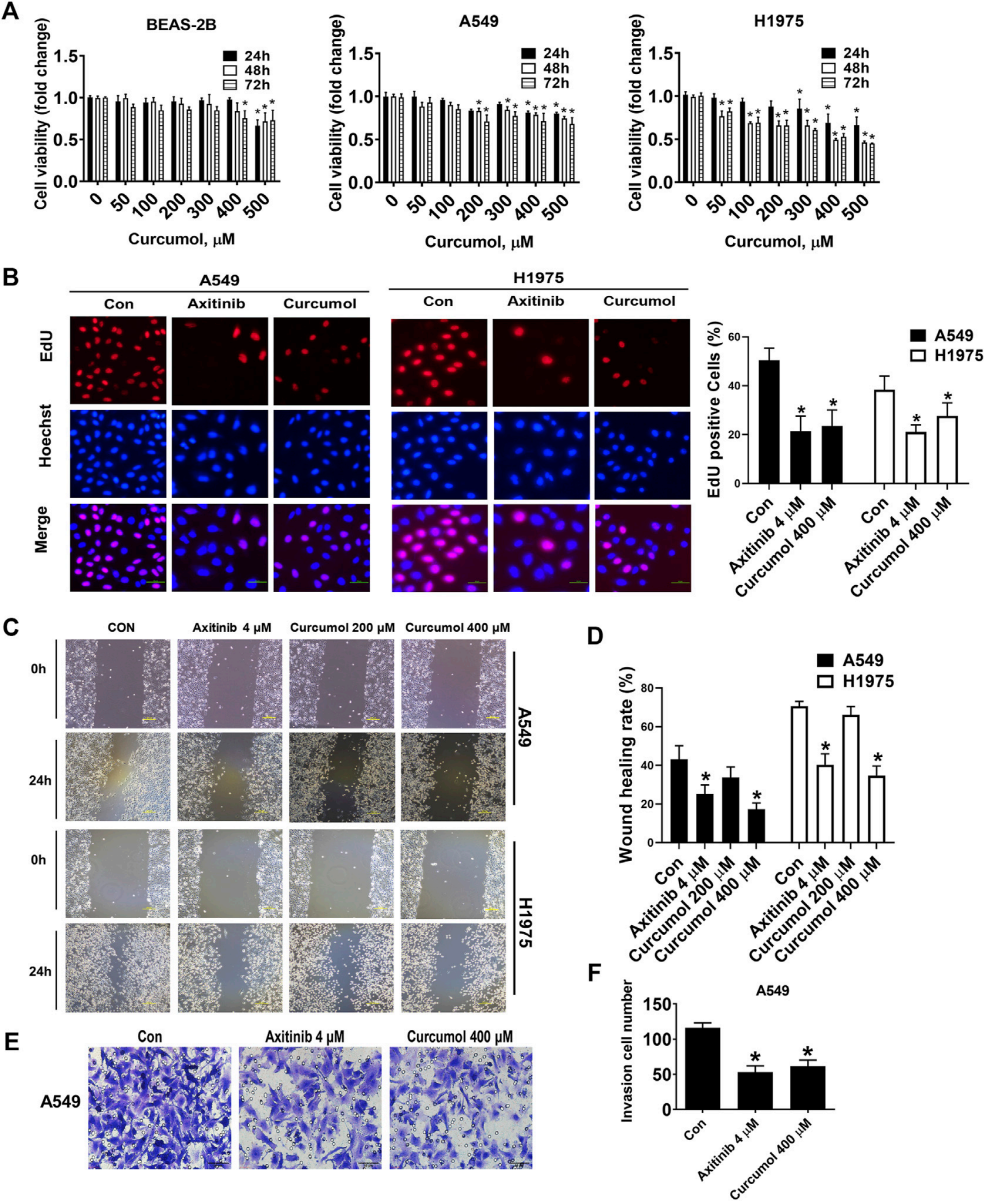
Curcumol attenuated NSCLC cells proliferation, migration, invasion capabilities. **(A)** Inhibitory effect of Curcumol on the cell viability of BEAS-2B, A549, H1975 cells at 24 h, 48 h and 72 h by MTT analysis. **(B)** A549 and H1975 cells were treated with Axitinib (4 μM) or Curcumol (400 μM) for 24 h, followed by EdU cell proliferation assay. Scale bar, 50 μm. **(C**,**D)** A549 and H1975 cells migration measured by wound healing assay. Scale bar, 200 μm. **(E**,**F)** A549 cells invasion ability examined by Transwell assays. Scale bar, 50 μm. The data are generated from five independent experiments (n = 5) and showed as mean ± SD. *p < 0.05, compared with control (0 μM or Con). NSCLC, non-small cell lung cancer.

At first, we evaluated the cytotoxicity of Curcumol against normal human bronchial epithelioid cells BEAS-2B cells to explore its safe concentration. Next, we evaluated inhibitory effect of Curcumol on the proliferation of Non-Small Cell Lung Cancer Cells, A549 and H1975. The results showed that Curcumol at the concentration of 400 μM at 24 h or 48 h was acceptable concentration and duration against BEAS-2B cells because it had limited cytotoxicity compared to that of vehicle control. A549 cells viability were reduced by 19.06%, 21.38%, 28.53%, when treated with Curcumol (400 μM) for 24 h, 48 h, 72 h respectively, compared to corresponding non-treated controls. The rate of cell proliferation was found to be significantly decreased (by 31.00%, 50.86% and 47.17%) in H1975 cells treated with Curcumol (400 μM), for 24 h, 48 h, and 72 h respectively, when compared to non-treated controls H1975 cells (Figure 1A). Meanwhile, EdU (Figure 1B) assay also showed that Curcumol could inhibit A549 and H1975 cell viability (EdU positive cells in Curcumol 400 μM group: A549 cells 23.58 ± 6.48%; H1975 cells 27.63 ± 5.34%, after normalization to control groups, shown as mean ± SD), with Axitinib (a commonly clinically used targeting anticancer drug) used as the positive control drug. Furthermore, we found that it also could attenuate the migration (wound healing rate in A549 cells: control group 43.16 ± 7.02%; Curcumol 400 μM group 17.28 ± 3.20%, H1975 cells: control group 70.57 ± 2.57%; Curcumol 400 μM group 34.70 ± 4.95%) (Figures 1C,D) and invasion (invasion cell number in A549 cells: control group 116 ± 7.07; Curcumol 400 μM group 61.40 ± 8.79) (Figures 1E,F) capability of NSCLC cells.

### A549 cell-derived conditioned medium affected HUVEC angiogenesis

**FIGURE 2:**
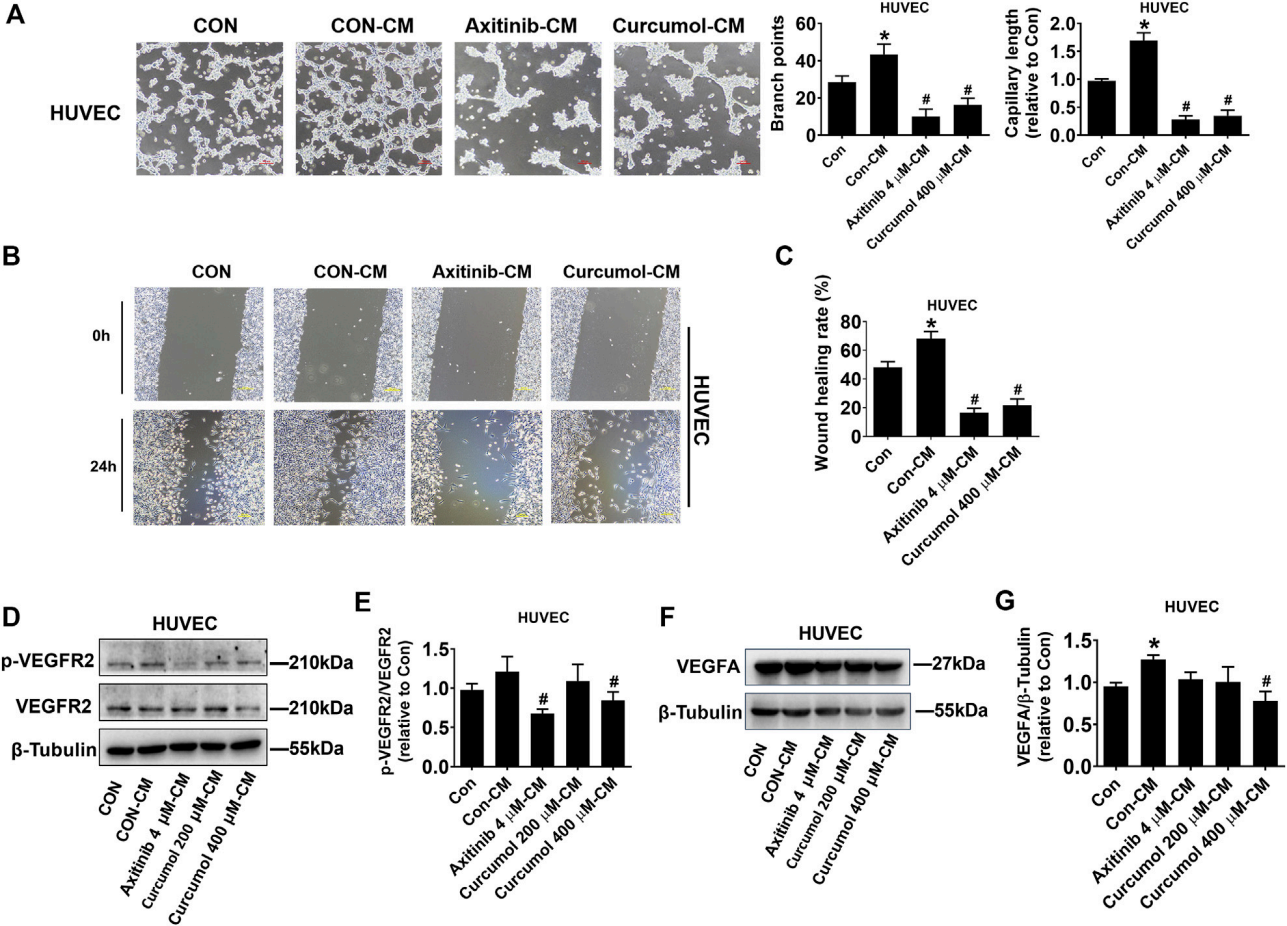
Effect of A549 cell-derived CM on HUVEC angiogenesis. HUVECs were cultured with CM derived from A549 for 24 h, then a series of assays, including **(A)** tube formation (Scale bar, 100 μm), **(B**,**C)** wound healing (Scale bar, 200 μm), **(D**–**G)** Western blot, were performed to investigate effect of Curcumol on endothelial cells in tumor microenvironment. CM, conditioned medium. HUVEC, human umbilical vein endothelial cell. The data are presented from five independent experiments (n = 5) run in triplicate. *p < 0.05, compared with control (Con). #*p* < 0.05, compared with Con-CM.

Curcumol is a monomer component of Chinese medicine *Curcuma*, and *Curcuma* is considered to have the effect of promoting blood circulation and removing blood stasis (Dosoky and Setzer, 2018). Therefore, we speculated that Curcumol may have a regulatory impact on angiogenesis. We treated A549 cells with Axitinib, Curcumol, or DMSO, then collected the conditioned medium (CM) for HUVEC culture (marked as Axitinib-CM, Curcumol-CM, Con-CM). We cultured HUVEC in these CMs and examined HUVEC tube formation (Figure 2A) and migration capacities (Figures 2B,C). Curcumol 400 μM-CM significantly inhibited HUVEC tube formation (branch points: Con-CM 43.33 ± 5.51; Curcumol 400 μM-CM 16.33 ± 3.51, fold change of capillary length: Con-CM 1.69 ± 0.14; Curcumol 400 μM-CM 0.35 ± 0.10, shown as mean ± SD) and migration, compared with Con-CM, which showed opposite effect. Then we checked the protein level of phosphorylated VEGFR2 and total VEGFR2, we found that phosphorylated/total VEGFR2 protein ratio was decreased in Axitinib-CM group (0.68 ± 0.05) and Curcumol 400 μM-CM group (0.84 ± 0.11), with Con-CM group (1.21 ± 0.19) as comparing object (Figures 2D,E). Moreover, the level of VEGFA expressed by HUVEC cultured with Curcumol 400 μM-CM (0.78 ± 0.12) was significantly down regulated, while up regulated in Con-CM group (1.27 ± 0.05) (Figures 2F,G). These data indicated that Curcumol affected A549 cells-induced VEGF pathway of endothelial cells in tumor microenvironment, modulating angiogenesis of HUVEC.

### Curcumol could regulate VEGFA, SP1 and mir-125b-5p expression

**FIGURE 3:**
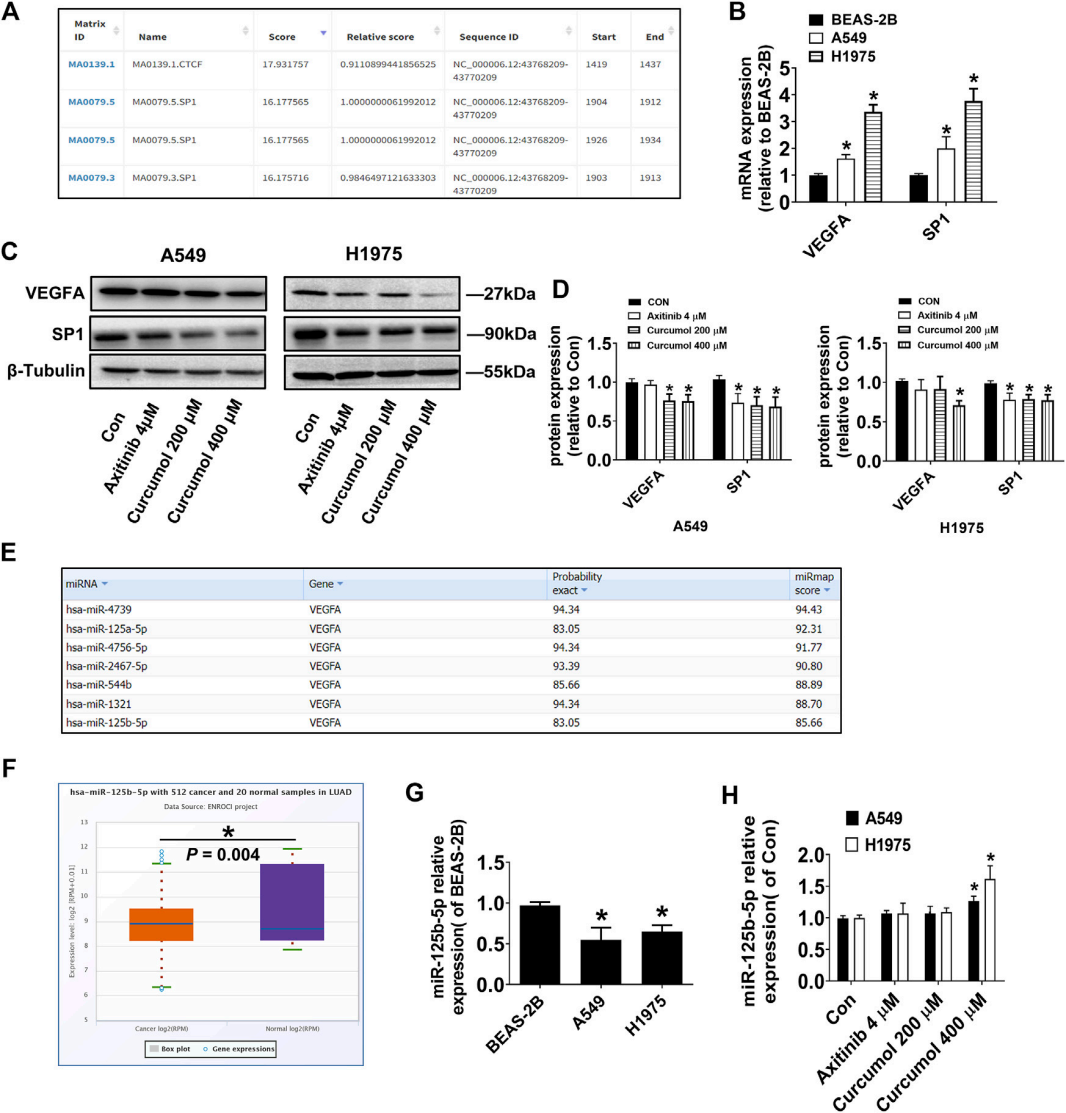
Curcumol decreased VEGFA, SP1, but increased miR-125b-5p Expression. **(A)** Transcription factors in descending order of score, that may regulate VEGFA transcription. **(B)** mRNA expression level of VEGFA and SP1 in A549 and H1975, relative to that of BEAS-2B. **(C**,**D)** VEGFA, SP1 protein expression levels were detected by Western blot. **(E)** MicroRNAs with more than seven base binding sites at the 3′UTR of VEGFA mRNA searched on miRmap. **(F)** Differential expression of miR-125b-5p in human normal tissues and tumor tissues. LUAD, Lung Adenocarcinoma. **(G)** Expression level of miR-125b-5p in A549 and H1975, relative to that of BEAS-2B. **(H)** Expression level of miR-125b-5p in A549 and H1975, tested by qRT-PCR. The data are presented from five independent experiments (n = 5) run in triplicate. *p < 0.05, compared with control (Con).

VEGFA around endothelial cells in the tumor microenvironment is partly derived from autocrine and mostly from paracrine of tumor cells. Therefore, we speculated that Curcumol may directly regulate the expression level of VEGFA in tumor cells. The transcription factors in descending order of score, that may regulate VEGFA transcription were predicted by software JASPAR (https://jaspar.genereg.net/). The list showed that SP1 (specificity protein 1) could be a good candidate (Figure 3A). qRT-PCR results showed that the mRNA levels of VEGFA and SP1 in lung cancer cells were significantly higher than that in normal lung epithelial cells (relative VEGFA mRNA level: BEAS-2B 1.00 ± 0.06; A549 1.62 ± 0.14; H1975 3.36 ± 0.27, relative SP1 mRNA level: BEAS-2B 1.00 ± 0.05; A549 2.09 ± 0.34; H1975 3.76 ± 0.46) (Figure 3B). Western blotting analysis results confirmed our assumptions, that Curcumol (400 μM) could decreased both VEGFA and SP1 protein level in A549 and H1975 cells (relative VEGFA protein level: A549 0.75 ± 0.08; H1975 0.71 ± 0.06, relative SP1 protein level: A549 0.69 ± 0.12; H1975 0.77 ± 0.07) (Figures 3C,D).

It is well known that microRNAs can degrade mRNA and reduce post transcriptional translation of target genes by pairing with the 3′ untranslated region of mRNA. We predicted the microRNAs with more than seven base binding sites at the 3′UTR of VEGFA mRNA by software miRmap (https://mirmap.ezlab.org/app/), and arranged them in descending order of score (Figure 3E). Next, the differential expression of those microRNAs in human normal tissues and tumor tissues was retrieved in the database ENCORI (https://starbase.sysu.edu.cn/). Interestingly, we found that miR-125b-5p was significantly lower in human lung adenocarcinoma tissues (Figure 3F), which was consistent with the results of cell experiments (relative miR-125b-5p expression level: BEAS-2B 0.97 ± 0.04; A549 0.55 ± 0.15; H1975 0.65 ± 0.08) (Figure 3G). Next, we tested whether Curcumol could affect the expression of miR-125b-5p in NSCLC cells and found that Curcumol (400 μM) could promote its expression (relative fold change of miR-125b-5p expression: A549 1.27 ± 0.08; H1975 1.62 ± 0.21) (Figure 3H).

### Overexpression of SP1 could upregulate VEGFA and downregulate mir-125b-5p

**FIGURE 4:**
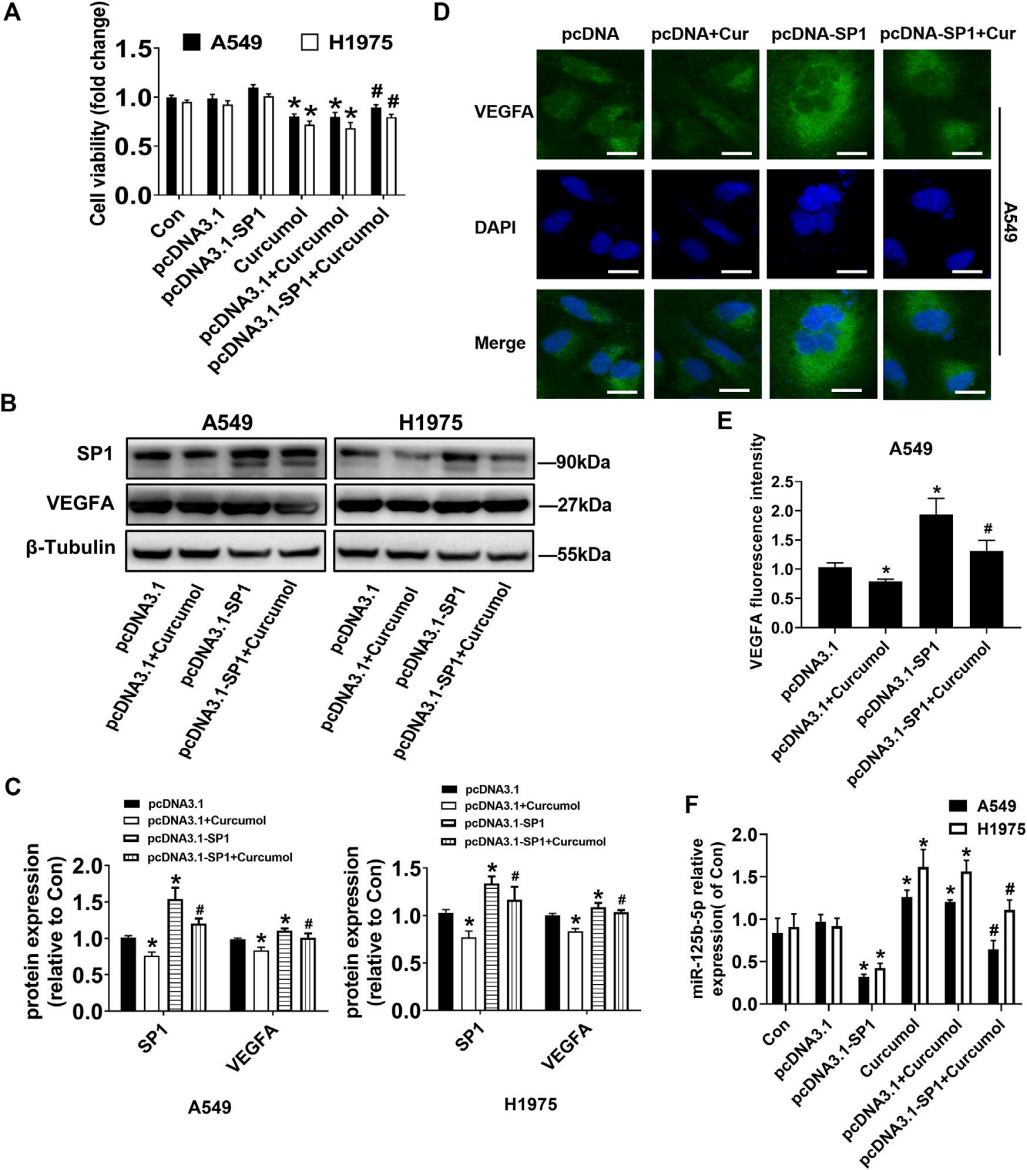
Overexpressed SP1 impacted Curcumol-mediated cell viability, VEGFA and miR-125b-5p. **(A)** A549 and H1975 were transfected with SP1 overexpression plasmid (pcDNA3.1-SP1 0.5 μg/ml) or control plasmid (pcDNA3.1) for 24 h, followed by added with Curcumol for another 24 h. Cell viability was calculated by OD (optical density) number of each well. **(B**,**C)** Protein expression level of SP1, VEGFA were detected by Western blot, with β-Tubulin as internal control. **(D**,**E)** The expression of VEGFA protein in A549 cells was detected by cellular immunofluorescence, and the localization and semi quantification were performed. DAPI was used to display nuclei. Scale bar, 20 μm. **(F)** NSCLC cells were transfected with vectors, followed by treating with Curcumol (final concentration 400 μM) for 24 h. Expression of miR-125b-5p were detected by qTR-PCR, with U6 as internal reference. The data are presented from five independent experiments (n = 5) run in triplicate. *p < 0.05, compared with control (Con). #*p* < 0.05, compared with pcDNA3.1 + Curcumol group.

To evaluate the underlying mechanisms associated with the anticancer and antiangiogenic effect of Curcumol, we applied transient transfection technique. SP1 overexpression vectors (pcDNA3.1-SP1) or control vectors (pcDNA3.1) were transfected into A549 and H1975 cells for up to 24 h, followed by treating with Curcumol for an additional 24 h. Overexpression of SP1 reversed the effect of Curcumol-inhibited cell viability of A549 and H1975 cells, determined by MTT assays (fold change of cell viability: in A549 cells pcDNA3.1 group 0.98 ± 0.04; pcDNA3.1 + Curcumol group 0.79 ± 0.05; pcDNA3.1-SP1 + Curcumol group 0.89 ± 0.03, in H1975 cells pcDNA3.1 group 0.92 ± 0.04; pcDNA3.1 + Curcumol group 0.68 ± 0.06; pcDNA3.1-SP1 + Curcumol group 0.80 ± 0.03) (Figure 4A). According to the previous prediction, we conjectured that SP1 might play a role in promoting the growth and angiogenesis of NSCLC cells by increasing the expression of VEGFA. Then, we explored whether SP1 could promote the expression of VEGFA protein through Western blot (Figures 4B,C) and immunofluorescence experiments (Figures 4D,E), and obtained positive results. Interestingly, qRT-PCR results suggested that SP1 could also attenuate the promoting effect of Curcumol on miR-125b-5p in NSCLC cells (fold change of miR-125b‐5p expression level: in A549 cells pcDNA3.1 group 0.97 ± 0.08; pcDNA3.1-SP1 group 0.32 ± 0.03; pcDNA3.1 + Curcumol group 1.20 ± 0.03; pcDNA3.1-SP1 + Curcumol group 0.64 ± 0.11, in H1975 cells pcDNA3.1 group 0.92 ± 0.09; pcDNA3.1-SP1 group 0.42 ± 0.06; pcDNA3.1 + Curcumol group 1.56 ± 0.13; pcDNA3.1-SP1 + Curcumol group 1.11 ± 0.12) (Figure 4F).

### MiR-125b-5p was able to influence VEGFA and SP1 expression *via* different ways

**FIGURE 5:**
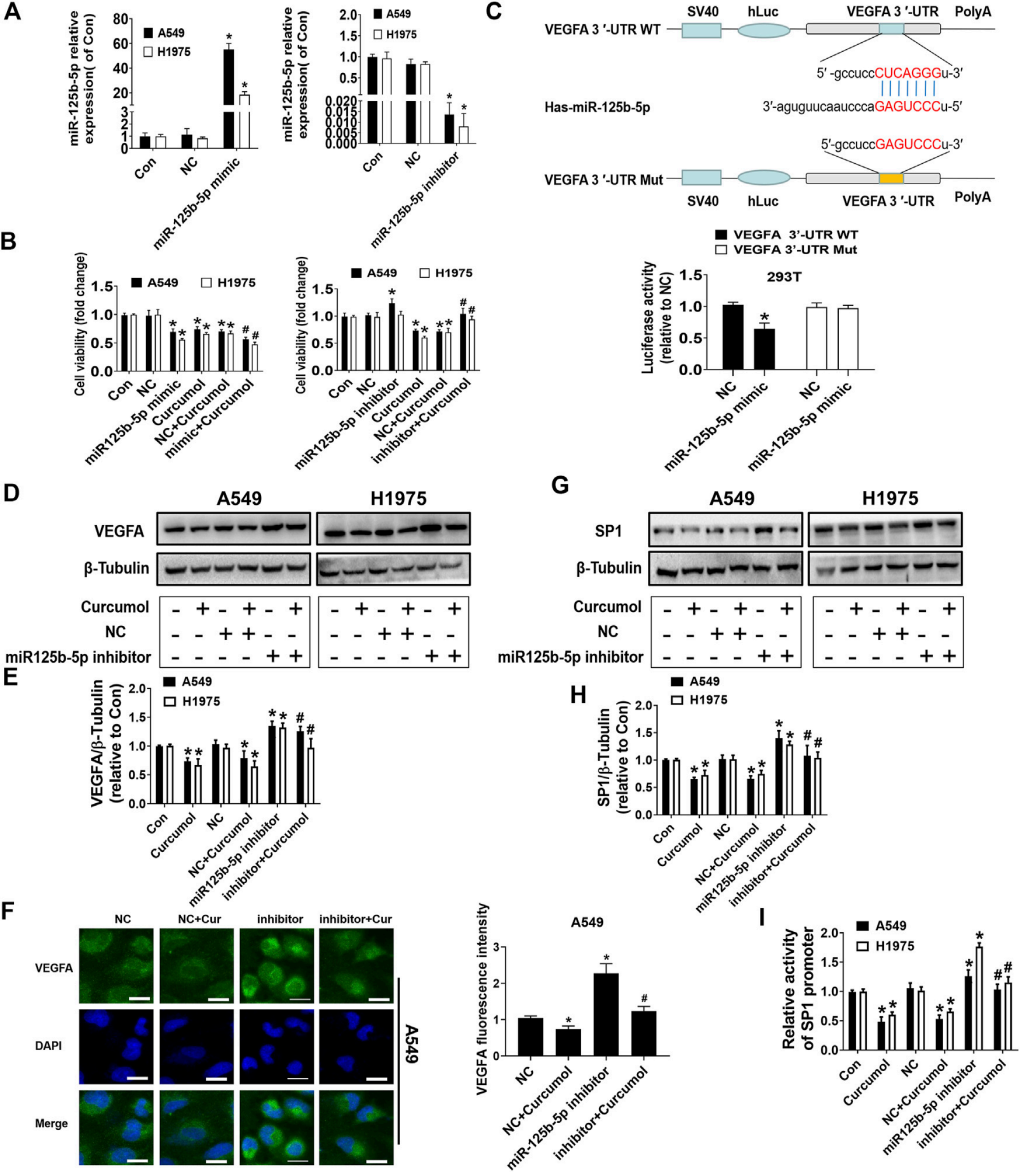
MiR-125b-5p mimics or inhititor modulated Curcumol-mediated cell viability, VEGFA and SP1 Expression. **(A**,**B)** Quantification of miR-125b-5p was shown after addition of mimics or inhibitor for 24 h. Subsequently, cell viability of A549 and H1975 were measured by MTT assay. **(C)** The luciferase reporter constructed with wild type or mutant VEGFA sequences were shown. 293T cells were transfected with VEGFA 3′UTR-WT or VEGFA 3′UTR-Mut vectors (0.5 μg/ml each well) for 24 h, then transfected with miR-125b-5p mimics (50 nM) or NC for another 24 h. The luciferase activity was detected on fluorescence microplate reader. VEGFA protein was displayed by **(D,E)**, Western blot analysis as well as **(F)** cellular immunofluorescence assay after transfection of miR-125b-5p inhibitor (50 nM) and addition of Curcumol (400 μM). Scale bar, 20 μm. **(G**,**H)** The bands of SP1 by Western blot analysis. β-Tubulin was used as internal control. **(I)** Dual-Luciferase Reporter Assay was carried out to detect SP1 promoter luciferase activity. The data are presented from five independent experiments (n = 5) run in triplicate. *p < 0.05, compared with control (Con). #*p* < 0.05, compared with NC + Curcumol group.

Next, we carefully explore the specific role and possible mechanism of miR-125b-5p in Curcumol-inhibited lung cancer cells growth. The results of MTT indicated that exogenous increase of miR-125b-5p level could promote the anti-cancer effect of Curcumol (fold change of cell viability: in A549 cells NC group 0.98 ± 0.09; miR-125b-5p mimic group 0.69 ± 0.05; NC + Curcumol group 0.70 ± 0.03; mimic + Curcumol group 0.56 ± 0.04, in H1975 cells NC group 1.00 ± 0.09; miR-125b-5p mimic group 0.56 ± 0.02; NC + Curcumol group 0.71 ± 0.05; mimic + Curcumol group 0.47 ± 0.04), while decreasing the level of miR-125b-5p could partially reverse the anti-cancer effect of Curcumol (fold change of cell viability: in A549 cells NC group 1.02 ± 0.04; miR-125b-5p inhibitor group 1.28 ± 0.08; NC + Curcumol group 0.72 ± 0.03; inhibitor + Curcumol group 1.04 ± 0.01, in H1975 cells NC group 0.99 ± 0.08; miR-125b-5p inhibitor group 1.02 ± 0.06; NC + Curcumol group 0.70 ± 0.07; inhibitor + Curcumol group 0.93 ± 0.06) (Figures 5A,B).

By utilizing multiple bioinformatics prediction databases, we acknowledged that miR-125b-5p has a relatively conservative binding site in the region of VEGFA mRNA 3′UTR. Subsequently, we created a pair of cDNA fragment of VEGFA containing the wild-type or mutated binding sites of miR-125b-5p. Then, dual-luciferase reporter assay was conducted to detect the luciferase activities, the results indicated that miR-125b-5p mimics significantly reduced the luciferase activities in 293T cells transfected with the wild-type cDNA fragment of VEGFA (1.03 ± 0.04), compared with negative control (NC) group (0.64 ± 0.09) (Figure 5C). Furthermore, miR-125b-5p inhibitor could rescue the inhibitory effect of Curcumol on VEGFA protein expression, verified by Western blot analysis (Figures 5D,E) as well as cell immunofluorescence assay (Figure 5F).

We wondered whether miR-125b-5p also has a regulatory effect on SP1, and if so, what is the possible mechanism. Surprisingly, miR-125b-5p was able to regulate SP1 protein expression (fold change of SP1 protein expression level: in A549 cells NC group 1.02 ± 0.07; NC + Curcumol group 0.66 ± 0.06; miR-125b-5p inhibitor group 1.40 ± 0.14; inhibitor + Curcumol group 1.08 ± 0.19, in H1975 cells NC group 1.01 ± 0.07; NC + Curcumol group 0.75 ± 0.07; miR-125b-5p inhibitor group 1.28 ± 0.06; inhibitor + Curcumol group 1.04 ± 0.11) (Figures 5G,H). Therefore, we continued to design dual-luciferase reporter assay to explore the effect of miR-125b-5p and Curcumol on SP1 promoter activity. At first, A549 and H1975 cells were all transfected with SP1 promoter plasmids, then co-transfected with miR-125b-5p inhibitor or NC, treated with Curcumol (400 μM) for additional 24 h. It turned out that Curcumol could decreased SP1 promoter activity, while miR-125b-5p inhibitor showed opposite effect (fold change of SP1 promoter activity: in A549 cells NC group 1.06 ± 0.09; NC + Curcumol group 0.53 ± 0.07; miR-125b-5p inhibitor group 1.26 ± 0.11; inhibitor + Curcumol group 1.04 ± 0.09, in H1975 cells NC group 1.01 ± 0.06; NC + Curcumol group 0.65 ± 0.05; miR-125b-5p inhibitor group 1.76 ± 0.07; inhibitor + Curcumol group 1.15 ± 0.10) (Figure 5I).

### Curcumol inhibited A549 cells xenograft tumor growth and reduced microvessel density

**FIGURE 6:**
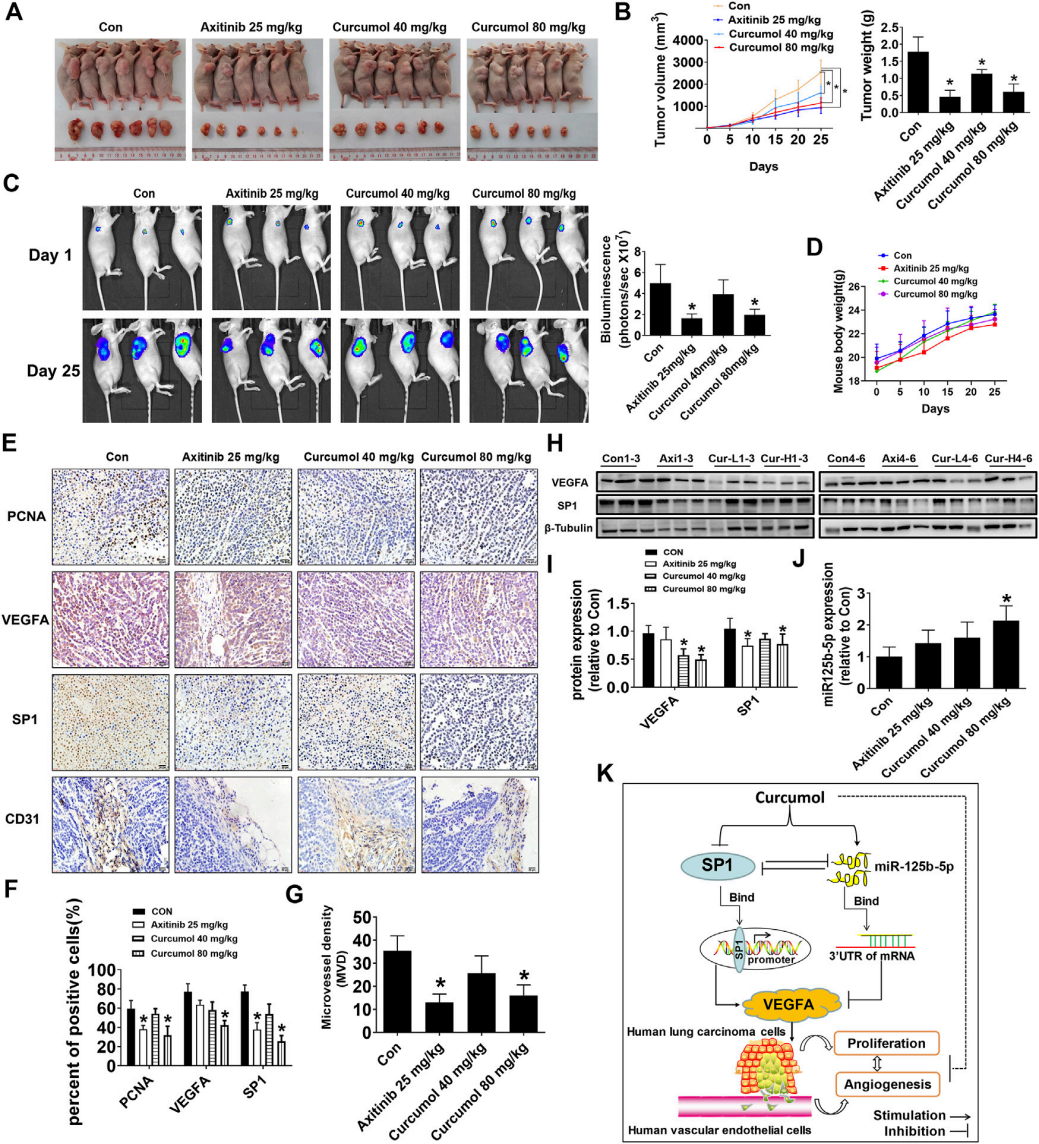
Curcumol reduced tumor burden and MVD of nude mice. **(A**,**B)** Mice (n = 6 per group) were divided into four groups (Con [saline], Axitinib [25 mg/kg], Curcumol [40 mg/kg], and Curcumol [80 mg/kg]). Tumor tissue were harvested and photographed after 25 days intragastric administration. Tumor volume and tumor weight were measured. **(C)** Tumor growth degree was evaluated by measuring bioluminescence signals in IVIS imaging system on 1st day and 25th day. **(D)** Body weight of mice in each group recorded every 5 days. **(E**–**G)** Immunohistochemistry (IHC) images of PCNA, VEGFA, SP1, CD31. Scale bar, 20 μm. ImageJ software was used to count positive cells. Microvessel density (MVD) was calculated through CD31 antibody staining. **(H**,**I)** VEGFA and SP1 protein expression level in mice fresh tumor tissue were detected by Western blot. Axi, Axitinib 25 mg/kg. Cur-L, Curcumol 40 mg/kg. Cur-H, Curcumol 80 mg/kg. **(J)** MiR-125b-5p expression level were detected by qTR-PCR, with U6 as internal control. **(K)** The diagram showed that Curcumol inhibited NSCLC cell growth and angiogenesis through suppressing SP1 expression, increasing miR-125b-5p expression, followed by downregulation of VEGFA. There is feedback regulatory axis and complex interaction network among these targets that together contribute to the inhibitory effect of Curcumol on NSCLC cells. *p < 0.05, compared with control (Con).

To further validate *in vitro* results, we constructed the xenograft tumor model with nude mice to test the antitumor effect of Curcumol. Luciferase-expressing A549 cells (A549-Luc) were injected subcutaneously to the right flank of each BALB/c nude mouse. We found that Curcumol could significantly suppress tumor volume (control group: 2544.20 ± 550.13 mm^3^; Curcumol 80 mg/kg group: 1146.35 ± 244.92 mm^3^), tumor weight (control group: 1.78 ± 0.43 g; Curcumol 80 mg/kg group: 0.61 ± 0.23 g) as well as tumor luciferase activity (control group: 4.99 ± 1.77; Curcumol 80 mg/kg group: 1.97 ± 0.54), compared with the control group (Figures 6A–C). It is worth noting that during the administration of the drug, nude mice were in good spirits in each group. There was no obvious abnormality in diet, defecation and urination, and no obvious adverse reactions were observed. The body weight was steadily increased, and there was no significant difference among the groups (Figure 6D).

Moreover, consistent with the *in vitro* results, decreased expression of SP1 and VEGFA protein was observed in both nude mice tumor tissues fixed with paraformaldehyde (Figures 6E,F) and fresh tumor tissues (Figures 6H,I). PCNA, which may well indicate cancer cell proliferation activity, also decreased within tumors from Curcumol 80 mg/kg group (control group: 59.41 ± 8.56%; Curcumol 80 mg/kg group: 32.03 ± 8.97%). Microvessel density (MVD) was calculated through CD31 antibody staining in mice tumor tissues (control group: 35.33 ± 6.51; Curcumol 80 mg/kg group: 16.00 ± 4.58) (Figure 6G). Last but not the least, miR-125b-5p expression increased apparently in Curcumol higher dose group (fold change of miR-125b-5p expression in control group: 1.00 ± 0.30; Curcumol 80 mg/kg group: 2.14 ± 0.46) (Figure 6J). Taken together, above results indicated that Curcumol may repress NSCLC cells growth and angiogenesis by regulating SP1/miR-125b-5p/VEGFA interaction network (Figure 6K).

## Discussion

Tumor microenvironment (TME), composed of various cells and extracellular components, plays a vital role in tumor initiation, angiogenesis, progression as well as metastasis. TME is also associated with cancer relapse after anticancer therapies (Ma L. et al., 2019; Rahma and Hodi, 2019; Hwang et al., 2020). Endothelial cells in TME are getting more and more attention for taking part in tumor development. Tumor cells physically interact with endothelial cells in TME and impact them by paracrine and juxtacrine signaling. Interestingly, endothelial cells in TME can also affect tumor cells by secreting angiocrine factors, such as biglycan, then promoting tumor cells metastasize. (Maishi and Hida, 2017). VEGFA is one of the major cytokines released from tumor cells to induce endothelial cells angiogenesis. In the present study, we focused on NSCLC cells secreted-VEGFA and its possible regulators’ impact on NSCLC cells proliferation and angiogenesis.

There were some studies on Curcumol anticancer effect as mentioned above, but whether it could potently interfere NSCLC cells growth was not sufficiently validated. Thus, we first underwent MTT assays to explore Curcumol role in NSCLC cells viability, with Axitinib as positive control (Supplementary Figure S1). We figured out both relatively safe dose and period, using BEAS-2B cells to evaluate. Subsequently, we found Curcumol could effectively hamper NSCLC cells proliferation, migration and invasion. Despite that Curcumol appeared to be able to increase the proportion of G0/G1 phase cells and decrease the proportion of S phase cells in A549, there was no statistical difference (Supplementary Figure S2). Then, we utilized A549-derived conditioned medium to mimic TME and cultured HUVECs with CM. Interestingly, HUVECs infiltrated in the blank CM (Con-CM) tended to form more branches, migrate faster, expressed more VEGFA than those in ordinary medium (Con). Moreover, Curcumol-CM group HUVECs showed impaired tube formation, migration, VEGFA expression. Collectively, Curcumol were able to modulate NSCLC cells development and angiogenesis *in vitro*.

Furthermore, we wondered if Curcumol could directly inhibit NSCLC cells express VEGFA, if so, what might be the upstream or downstream molecules. By referring to renowned transcription factor prediction database JASPAR, we chose SP1 as a candidate. In breast cancer cells, zinc finger E-box binding homeobox 1 (ZEB1) activated VEGFA transcription through enhancing SP1 recruitment to VEGFA promoter (Liu et al., 2016). Another study confirmed the direct interaction between VEGFA and SP1 in breast cancer cells by Chromatin Immunoprecipitation (ChIP) experiment (Li et al., 2020). In our study, VEGFA and SP1 transcription level in NSCLC cells were simultaneously upregulated in contrast to BEAS-2B and Curcumol could decrease both protein level. Since mircroRNAs plays critical part in cellular biology process, we searched miRNA-mRNA binding site prediction website as well as differential expression data between NSCLC and normal samples from human specimen, which were extracted from The Cancer Genome Atlas (TCGA) project. Therefore, we put minds on miR-125b-5p, whose expression level was downregulated both in human NSCLC samples and cells. Surprisingly, Curcumol could promote miR-125b-5p expression in both A549 and H1975 cells.

We then discovered how SP1, miR-125b-5p functioned in Curcumol-mediated NSCLC cells growth and VEGFA expression individually. The results were in line with our assumption, that overexpression of SP1 could neutralize inhibitory effect of Curcumol on NSCLC cells. SP1 overexpression plasmids could also reduce miR-125b-5p level, displaying opposite outcome from Curcumol, of which the mechanism was worth deliberating. When it came to miR-125b-5p, there was one study demonstrating that miR-125b-5p expression was downregulated in heat-denatured dermal tissues and it showed negative correlation with VEGFA (Zhou et al., 2021). Our study also identified there was conservative binding site for miR-125b-5p in the 3′UTR of VEGFA sequence. Mimics and inhibitors of miR-125b-5p turned out to interfere NSCLC cells growth towards different direction. Apart from effect on VEGFA expression, miR-125b-5p inhibitor could resist inhibition impact of Curcumol on SP1 protein, maybe by modulating SP1 promoter activity.

Moreover, our *in vivo* data was consistent with *in vitro* data, further validating the anticancer and anti-angiogenesis of Curcumol on NSCLC xenograft tumors *via* regulation of SP1, miR-125b-5p and VEGFA expression. However, there are also some limitations about this study. For example, HUVECs are large blood vessel endothelial cells (ECs), which although a representative of ECs (and is used widely in studies) behaves quiet differently from ECs of the microvasculature such as those seen in growing tumors. We will perform further study in representative microvascular endothelial cells, like HMEC-1, to reconfirm our hypothesis.

## Conclusion

The present study showcased that Curcumol can inhibit NSCLC cells proliferation and angiogenesis, which were associated with regulation of VEGFA by SP1 and miR-125b-5p. What’s more, there was interaction between SP1 and miR-125b-5p in NSCLC cells. These findings enhanced our understanding of possible mechanism by which Curcumol prevented NSCLC cells progression, may provide novel molecular targets for NSCLC therapies as well.

## Data Availability

The datasets presented in this study can be found in online repositories. The names of the repository/repositories and accession number(s) can be found in the article/Supplementary Material.

## Abbreviations

NSCLC, non-small cell lung cancer; TME, tumor microenvironment; HUVECs, human umbilical vein endothelial cells; SP1, specificity protein 1; ROS, reactive oxygen species; VEGFR2, vascular endothelial growth factor receptor 2; VEGFA, vascular endothelial growth factor A; 3′UTR, 3′ untranslated region; DMSO, dimethyl sulfoxide; MTT, 3‐ (4,5‐dimethylthiazol‐2‐yl) ‐2,5‐diphenyltetrazolium bromide; OD, optical density; EdU, 5-Ethynyl-2′-deoxyuridine; CM, conditioned medium; PVDF, polyvinylidene fluoride; qRT‐PCR, real‐time quantitative polymerase chain reaction; NC, negative control; MVD, microvessel density; ZEB1, zinc finger E-box binding homeobox 1.
