# Supplementary material for: Curcumol repressed cell proliferation and angiogenesis via SP1/mir-125b-5p/VEGFA axis in non-small cell lung cancer
Source: Front Pharmacol. 2022 Nov 18;13:1044115. doi: 10.3389/fphar.2022.1044115 (PMC9716069; doi:10.3389/fphar.2022.1044115)
Supplement: Supplementary file 1 [file DataSheet1.PDF]

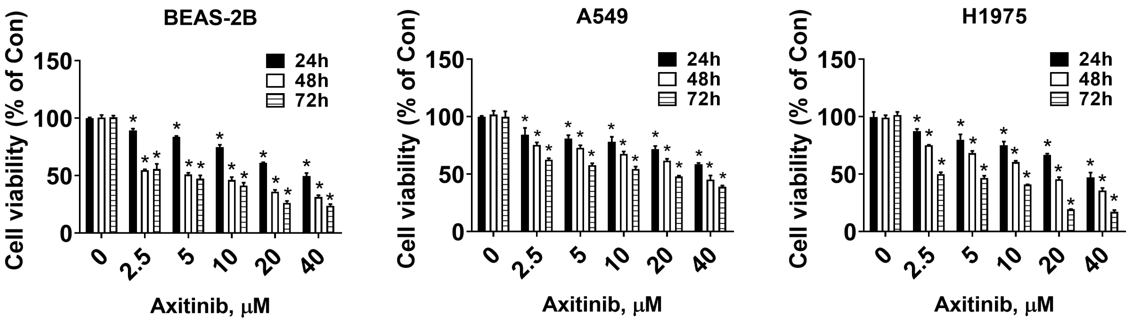

FIGURE S1 | Axitinib inhibited cell growth of BEAS-2B, A549 and H1975. *$p < 0.05$, compared with control (0 μM).

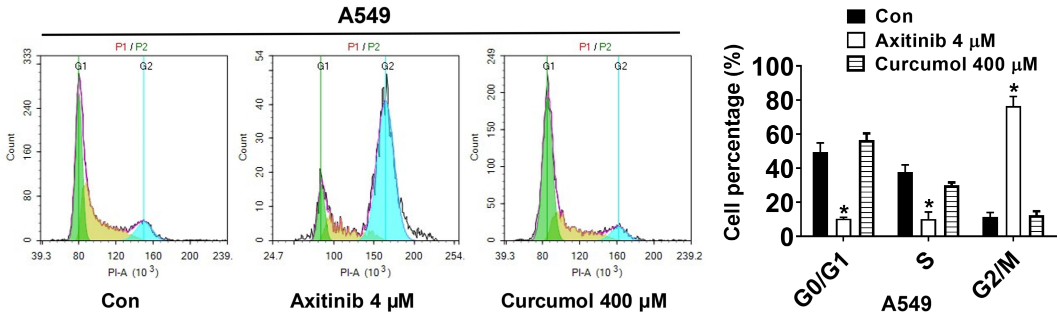

FIGURE S2 | Effect of Curcumol and Axitinib on cell cycle in A549 cells. *$p < 0.05$, compared with control (Con).
